# Magnetic resonance temporal diffusion tensor spectroscopy of disordered anisotropic tissue

**DOI:** 10.1038/s41598-018-19475-y

**Published:** 2018-02-13

**Authors:** Jonathan Scharff Nielsen, Tim B. Dyrby, Henrik Lundell

**Affiliations:** 1 Danish Research Centre for Magnetic Resonance, Centre for Functional and Diagnostic Imaging and Research, Copenhagen University Hospital Hvidovre, Copenhagen, Denmark; 2 Department of Applied Mathematics and Computer Science, Technical University of Denmark, Kongens Lyngby, Denmark

## Abstract

Molecular diffusion measured with diffusion weighted MRI (DWI) offers a probe for tissue microstructure. However, inferring microstructural properties from conventional DWI data is a complex inverse problem and has to account for heterogeneity in sizes, shapes and orientations of the tissue compartments contained within an imaging voxel. Alternative experimental means for disentangling the signal signatures of such features could provide a stronger link between the data and its interpretation. Double diffusion encoding (DDE) offers the possibility to factor out variation in compartment shapes from orientational dispersion of anisotropic domains by measuring the correlation between diffusivity in multiple directions. Time dependence of the diffusion is another effect reflecting the dimensions and distributions of barriers. In this paper we extend on DDE with a modified version of the oscillating gradient spin echo (OGSE) experiment, giving a basic contrast mechanism closely linked to both the temporal diffusion spectrum and the compartment anisotropy. We demonstrate our new method on post mortem brain tissue and show that we retrieve the correct temporal diffusion tensor spectrum in synthetic data from Monte Carlo simulations of random walks in a range of disordered geometries of different sizes and shapes.

## Introduction

Diffusion weighted imaging (DWI) probes the mobility of water molecules and provides a high sensitivity to tissue composition on length scales far below the image resolution of conventional magnetic resonance imaging (MRI). Since its introduction in the 1980’s it has become a popular imaging biomarker for detection of subtle differences between healthy and diseased tissue with important clinical application in ischemic stroke and tumor classification^1^. DWI data from conventional pulsed field gradient spin echo (PGSE) experiments is commonly approximated by a diffusion tensor, i.e. *diffusion tensor imaging* (DTI), to provide rotationally invariant metrics characterizing the diffusion in anisotropic tissue compartments^2^. Besides mean diffusivity reflecting cellular packing and size, DTI provides anisotropy estimates quantified by fractional anisotropy (FA) which is a characteristic feature of anisotropic cell types such as axons in white matter and muscle fibers^3^. An interpreter of DTI data must consider a number of effects operating on microscopic to macroscopic length scales. These effects range from direct influences on the water mobility such as cell sizes and shapes, to ensemble effects from orientational dispersion or partial volumes of differing tissue types and cerebrospinal fluid (CSF). Multiple relaxation rates or exchange between compartments can also affect the signal depending on the time frame of the experiment^4^.

### Model based approaches

A number of biophysical models for extracting microscopic tissue properties from conventional DWI data acquired with pulsed field gradient spin echo (PGSE) experiments have been proposed and can guide this interpretation^5,6^. Brain tissue is often modelled using thin tubes or 1D sticks to capture the mainly fibrous intracellular component of axons, dendrites or astrocytes embedded in an extracellullar space described by a time independent diffusion tensor^7,8^. Popular models like NODDI (Neurite Orientation Dispersion and Density Imaging) provide estimates of neurite density and orientational dispersion that may also be extracted by model guided interpretation of DTI metrics^9,10^. The DWI signal is in such models usually assumed to arise from separate compartments with each its diffusion tensor, making the DWI signal a sum of mono-exponential decays with respect to diffusion weighting. In reality, non-monoexponential effects arise in the signal either from compartment mixtures, time dependent diffusion or dispersion of anisotropic compartments. This poses a complex inverse problem where many different qualities provide similar features in the measured data resulting in a broad variety of plausible interpretations^11,12^. Alternative models describing exchange across the heterogeneity is a different approach for describing the complexity of real tissue^6^. In this context, basic model parameters such as the single compartment diffusion coefficients are yet pending to be quantitatively verified and understood.

### Need for data driven specificity and model independence

As important as understanding the biophysical interpretation of the model parameters is to understand how they vary over a healthy population or in specific pathologies and how such variations may affect the model predictions and their interpretations. Model free approaches like kurtosis imaging offer an alternative approach to representing non-monoexponential features in DWI data unbiased by model assumptions, but does not solve the problem of low specificity^13,14^. Sensitizing the data acquisition itself to correlations between multiple parameters is one way to achieve a richer information content from the sample and could be a way forward to improve specificity to individual signal components.

### Double Diffusion Encoding (DDE)

Double diffusion encoding (DDE) or multidimensional diffusion encoding (MDE) are approaches where the signal is weighted to the correlation in diffusivity between multiple independent directions within the same excitation^15^. This allows differentiation of the signal from a distribution of disperse anisotropic compartments from that of a distribution of isotropic compartments with varying (isotropic) diffusivities^15–17^. Different implementations of this principle have recently been developed suitable for imaging and the derived microscopic FA (*μ*FA) is equivalent to FA but insensitive to global effects of dispersion and thus proposed as a more specific marker of tissue microstructure^18–20^.

### Oscilating Gradient Spin Echo (OGSE) and time dependent diffusion

Diffusion time is an additional conceptually interesting parameter for probing the microstructure at different length scales^21–24^. Time dependent diffusivity has been shown to arise in biophysical models that include distributions of permeable barriers^25^, from general random perturbations to the local diffusivity^22^ and from diffusion between impermeable barriers^26^. In neuronal tissue, diffusion time is a weak contrast parameter on the time scales reachable with conventional PGSE in a clinical setting^27–29^ but shorter time scales can be investigated with oscillating gradient spin echo (OGSE)^30–32^. Time dependence measured with short diffusion times with PGSE or high frequencies with OGSE are associated^6,22^. However, OGSE may provide a higher diffusion weighting for given gradient hardware limitations. Moreover, exchange and mixing during a longer OGSE gradient duration will provide a closer to mono-exponential signal attenuation as the individual spins have probed a larger portion of the whole geometry^33,34^. A cosine modulated diffusion weighting gradient train is a frequency specific filter for the temporal diffusion spectrum (*D*(*ω*)) which provides a stringent description of a restricted diffusion compartment that in turn can be used to describe the signal from arbitrary gradient schemes^26,35^. *D*(*ω*), formally the Fourier transform of the molecular velocity autocorrelation function^26,36^, describes the transition from low frequencies, where reflections at barriers cause temporal correlations in molecular displacement, to high frequences where the diffusion is free and Brownian. OGSE measurements can be used to eg. characterize different tumors and ischaemic tissue^32,37,38^, and provides a strong tissue contrast in brain regions with large cell densities such as hippocampus and cerebellum^39–41^.

### Combining DDE and OGSE: Circularly Polarised OGSE (CP-OGSE)

Requiring strong gradient amplitudes, OGSE is mainly explored in post mortem settings on powerful preclinical MRI systems, but practical implementations have also been demonstrated for human *in vivo* use^38,42^. We recently introduced circularly polarised OGSE (CP-OGSE) with the main benefit of improving the contrast to noise by increasing the diffusion weighting of the conventional OGSE experiment with a factor of two^41^. CP-OGSE applies two orthogonal and dephased OGSE gradient encoding trains providing independent time dependent diffusion weighting in two directions simultaneously, displaying both the qualities of a DDE experiment as earlier proposed^41^.

### Extending CP-OGSE: Eliptically Polarised OGSE (EP-OGSE)

In this work we demonstrate the possibility of mapping the diffusion spectrum in disordered samples with a modified version of CP-OGSE we call *elliptically polarised OGSE* (EP-OGSE) by modulating the relative strengths and phases of two orthogonal oscillating gradients. We show that this approach gives a direct contrast to microscopic and time dependent anisotropy in disordered samples with an isotropic orientational distribution of separate tissue compartments and how the frequency dependent microscopic (single compartment) DTI metrics can be retrieved. We demonstrate this effect in EP-OGSE data acquired from an *ex vivo* monkey brain, quantifying the frequency dependent anisotropic diffusion tensors in gray and white matter. Monte Carlo simulations confirm our theoretical predictions, and resolve single compartment diffusion tensor spectra in disordered samples.

## Theory

### Gradient waveform and signal attenuation

The diffusion weighting gradient vector in conventional PGSE assumes a parallel and anti-parallel configuration in the gradient train. In OGSE, it follows a cosine trajectory. In CP-OGSE, two perpendicular out of phase gradients simultaneously follow a cosine trajectory, making the gradient trajectory circular. The EP-OGSE waveform used in this study alters the eccentricity of this circular trajectory via the ellipticity angle *χ*. An idealised representation of this gradient waveform is:

1 $${\bf{g}}(t)=\{\begin{array}{rl}G\,\cos (\chi )\cos ({\omega }_{m}t)\hat{{\bf{x}}} & {\rm{if}}\,t\le \frac{\pi }{2{\omega }_{m}}\\ G\,\cos (\chi )\cos ({\omega }_{m}t)\hat{{\bf{x}}}+G\,\sin (\chi )\sin ({\omega }_{m}t)\hat{{\bf{y}}} & {\rm{if}}\,\frac{\pi }{2{\omega }_{m}} < t\le T,\\ G\,\sin (\chi )\sin ({\omega }_{m}t)\hat{{\bf{y}}} & {\rm{if}}\,T < t\le T+\frac{\pi }{2{\omega }_{m}}\mathrm{.}\end{array}$$

where *G* is the maximum gradient amplitude, *ω*_*m*_ is the angular modulation frequency, *T* is the duration of each gradient. $\hat{{\bf{x}}}$ and $\hat{{\bf{y}}}$ are unit vectors. Under the Gaussian phase approximation (GPA)^1,36,43^ absent bulk motion and in a single tissue compartment, the DWI signal *E* from a single gradient trajectory at readout time ${\tau }=T+\frac{\pi }{2{\omega }_{m}}$ follows:

2 $$E={{\rm{e}}}^{-\beta }$$

3 $$\beta =\frac{1}{2\pi }{\int }_{-\infty }^{\infty }{\bf{F}}(\omega ){\bf{D}}(\omega ){{\bf{F}}}^{{\rm{T}}}(-\omega )d\omega \,{\rm{with}}\,{\bf{F}}(\omega )={\int }_{0}^{\tau }{\bf{F}}(t){{\rm{e}}}^{i\omega t}dt\,{\rm{and}}\,{\bf{F}}(t)=\gamma {\int }_{0}^{t}{\bf{g}}(t^{\prime} )dt^{\prime} ,$$

where *β* is the spin phase variance over the ensemble of water molecules, here related to the frequency dependent diffusivity tensor **D**(*ω*) and *γ* is the gyromagnetic ratio^26,36^. The signal is effectively characterized by **D**(*ω*) filtered by ***F***(*ω*)***F***^Τ^(−*ω*). Under GPA and encoding spectra with their weight dominated by a single peak at the oscillation frequency *ω*_*m*_ we can probe **D**(*ω*) with the signal attenuation factor *β* = Σ*B*_*ij*_(*ω*_*m*_)D_*ij*_(*ω*_*m*_) in Eqn. 2 where ***D***_*ij*_ and ***B***_*ij*_ are the respective elements of **D**(*ω*) and the frequency specific diffusion weighting **B**(*ω*_*m*_) matrix^2,39^. The latter is for the gradient trajectory in Eqn. 1 then given by:

4 $${\bf{B}}({\omega }_{m})={\int }_{0}^{T}{\bf{F}}(t){{\bf{F}}}^{{\rm{T}}}(t)dt=\frac{{\gamma }^{2}{G}^{2}T}{2{\omega }_{m}^{2}}[\begin{array}{ccc}{\cos }^{2}(\chi ) & \frac{2\pi \,\cos (\chi )\sin (\chi )}{T{\omega }_{m}} & 0\\ \frac{2\pi \,\cos (\chi )\sin (\chi )}{T{\omega }_{m}} & {\sin }^{2}(\chi ) & 0\\ 0 & 0 & 0\end{array}]$$

**Figure 1:**
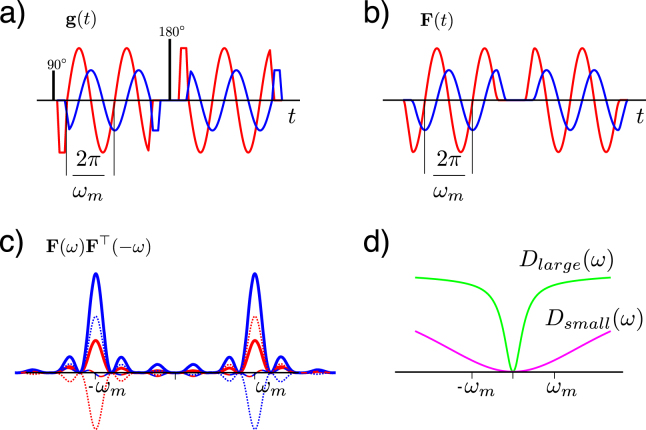
(**a**) An example of two orthogonal gradient components of an elliptically oscillating gradient vector with ellipticity angle *χ* = 30° and modulation frequency *ω*_*m*_. The waveforms shown are the effective gradients with inverted polarity after the 180°-pulse in this spin echo experiment. (**b**) The dephasing vector components calculated as the time integral of the gradients in a). (**c**) The weighting filter of the diffusion spectrum tensor is given by the elements of **F**(*ω*)**F**^Τ^(−*ω*). The diagonal elements are real with the same distinct peak at the modulation frequency in both axes but with modulated strengths (solid lines). The off diagonal elements are complex (real (dashed), imaginary (dotted)) but integrate to zero. (**d**) Examples of temporal diffusion spectra of large and small restrictions. The gradient trajectory in (**a**) would mainly filter out the diffusivity around *ω*_*m*_.

The full **D**(*ω*) tensor can be estimated with a DTI approach by acquiring a range of measurements with different **B**-matrices realized with different gradient strengths and orientations and this carries over to EP-OGSE where *χ*, *G*, *T*, *ω*_*m*_, $\hat{{\bf{x}}}$ and $\hat{{\bf{y}}}$ are the free parameters. This allows probing of the diffusion spectrum, leading to curves like on Fig. 1d, with information about restrictions in the tissue. The off diagonal terms in **B**(*ω*_*m*_) both rotate the elliptical polarization in the *xy*-plane and shift the eigenvalues, corresponding to unwanted gradient interactions. For the idealized gradient representation in Eqn. (1), their magnitude relative to the diagonals however vanishes as $\frac{1}{{\omega }_{m}}$ ((4)), implying that their influence is constrained to low frequencies. They are further proportional to the assymmetric function cos(*χ*)sin(*χ*), which is a feature of the *χ*-modulation that carries over to practical EP-OGSE implementations. The off diagonal terms can thus be eliminated by applying the first and second gradient trajectories in a spin echo sequence with opposed rotations, i.e. *χ*_1_ = −*χ*_2_. For the diagonalized **B**(*ω*_*m*_)-matrix, the attenuation can now be written as:

5 $$\beta =b[{D}_{xx}({\omega }_{m}){\rm{c}}{\rm{o}}{{\rm{s}}}^{2}\chi +{D}_{yy}({\omega }_{m}){\rm{c}}{\rm{o}}{{\rm{s}}}^{2}\chi ]$$

where *b* is the total diffusion weighting determined by the trace of Eqn. 4, $b=\frac{{\gamma }^{2}{G}^{2}T}{2{\omega }_{m}^{2}}$.

### Modulating the polarisation and parctical EP-OGSE implementation

The polarisation angle *χ* alters the shape of the elliptical polarisation. *χ* = 0° corresponds to linear polarisation in the x-direction, *χ* = 90° corresponds to linear polarisation in the y-direction and *χ* = 45° corresponds to circular polarisation (CP-OGSE)^41^. Several practical considerations come into the design of OGSE gradients, in particular the initiation of the gradient trains and the polarity of the gradient after the refocusing pulse^35,42^. We will in the following simulations and experiments use orthogonal gradient pairs as previously implemented in ref.^41^ but independently scaled by varying *χ*. Figure 1a shows the actual gradients used for *ω*_*m*_ = 100 Hz parametrized with respect to an intermediate *χ* = 30°. The filters acting on **D**(*ω*_*m*_) are from Eqn. 3 given by the elements of **F**(*ω*)**F**^Τ^(−*ω*) and shown in Fig. 1c. Note that the diagonal elements (solid lines) have identical shape with two symmetric peaks at *ω* and −*ω* but scaled according to *χ*. The sharpness of those peaks increases with the number of oscillations. The off diagonal terms are complex with a negligible real part (dashed lines), a consequence of the spin echo sequence with opposed rotations, and an imaginary part (dotted lines) with odd symmetry which thus integrate to zero in Eqn. 3. Since the *b*-value for fixed *T* decreases quickly with frequency as an inverse square, the gradient strength is typically adjusted to match a constant *b*-value over a range of *ω*_*m*_.

### EP-OGSE signal in ensembles of disordered axially symmetric compartments

From the signal attenuation of a single compartment we now consider ensembles of non-exchanging microscopic compartments. With EP-OGSE under the GPA, the attenuation from a single non-exchanging compartment is monoexponential in *b* at fixed *ω*_*m*_ (Eqns (2) and (5)). A parametrization of the signal that satisfies this and is both appropriate for cylindrically symmetric geometries and amenable to spherical averaging is considered in ref.^44^. The attenuation with a gradient along an axis $\hat{{\bf{a}}}$ for a compartment oriented along axis ***n*** is here modelled as:

6 $${\beta }_{a}={b}_{a}{({\bf{n}}\cdot \hat{{\bf{a}}})}^{2}{D}_{L}+b\mathrm{[1}-{({\bf{n}}\cdot \hat{{\bf{a}}})}^{2}]{D}_{T},$$

where *D*_*L*_ and *D*_*T*_ are the frequency specific diffusivities in respectively the longitudinal and transversal axes of the compartment and *b*_*a*_ is diffusion weighting along $\hat{{\bf{a}}}$. For a gradient train in the xy-plane it becomes:

7 $$\begin{array}{c}{\beta }_{x}=b({(\hat{{\bf{n}}}\cdot {\bf{x}})}^{2}{D}_{L}+[1-{(\hat{{\bf{n}}}\cdot \hat{{\bf{x}}})}^{2}]{D}_{T}){\cos }^{2}\chi =b\cdot ({D}_{L}{\sin }^{2}\theta {\cos }^{2}\phi \\ \,\,\,\,\,\,\,\,\,\,+\,{D}_{T}({{\rm{c}}{\rm{o}}{\rm{s}}}^{2}\theta {{\rm{c}}{\rm{o}}{\rm{s}}}^{2}\phi +{{\rm{s}}{\rm{i}}{\rm{n}}}^{2}\phi )))\cdot {{\rm{c}}{\rm{o}}{\rm{s}}}^{2}\chi \end{array}$$

8 $$\begin{array}{c}{\beta }_{y}=b({(\hat{{\bf{n}}}\cdot {\bf{y}})}^{2}{D}_{L}+[1-{(\hat{{\bf{n}}}\cdot \hat{{\bf{y}}})}^{2}]{D}_{T}){\sin }^{2}\chi =b\cdot ({D}_{L}{\sin }^{2}\theta {\sin }^{2}\phi \\ \,\,\,\,\,\,\,\,\,+\,{D}_{T}({\cos }^{2}\theta {\sin }^{2}\phi +{\cos }^{2}\phi )))\cdot {\sin }^{2}\chi \end{array}$$

9 $$E^{\prime} ={{\rm{e}}}^{-{\beta }_{x}-{\beta }_{y}}\mathrm{.}$$

Here, *θ* and *φ* denote the azimuthal and polar angles of the compartment orientation vector ***n*** in spherical coordinates.

The signal from an ensemble of compartments with an isotropic orientational distribution becomes:

10 $$E=\frac{1}{4\pi }{\int }_{-\pi }^{\pi }{\int }_{-\pi \mathrm{/2}}^{\pi \mathrm{/2}}E^{\prime} \cdot \,\sin \,\theta d\theta d\phi $$

The integral in Eqn. 10 can for linear and circular polarizations be written in forms independent of the azimuth *φ* and performed:

11 $${E}_{\chi {\mathrm{=0}}^{\circ }}=\sqrt{\pi }{e}^{-b{D}_{T}}\frac{erf(\sqrt{b({D}_{L}-{D}_{T})})}{\mathrm{2\ }\sqrt{b({D}_{L}-{D}_{T})}}$$

12 $${E}_{\chi {\mathrm{=45}}^{\circ }}=\sqrt{\pi }{e}^{-b\mathrm{/2(}{D}_{T}+{D}_{L})}\frac{erf(\sqrt{b\mathrm{/2(}{D}_{T}-{D}_{L})})}{\mathrm{2\ }\sqrt{b\mathrm{/2(}{D}_{T}-{D}_{L})}}$$

A uniform distribution can be emulated in data from samples with unknown orientational distributions by a powder average, i.e. averaging measurements with uniformly distributed gradient directions^19,45^. Variations of Eqn. 11 have been used in several applications in diffusion weighted imaging and spectroscopy^8,44,46,47^. A general solution to these equations in the special case of axially symmetric diffusion weighting and diffusion tensors is derived in ref.^48^ From those estimates of *D*_*L*_ and *D*_*T*_ the single compartment *μ*FA can be calculated as:

13 $$\mu FA=\frac{{|}_{T}{D}_{L}-D|}{\sqrt{{D}_{L}^{2}+2{D}_{T}^{2}}}$$

**Figure 2:**
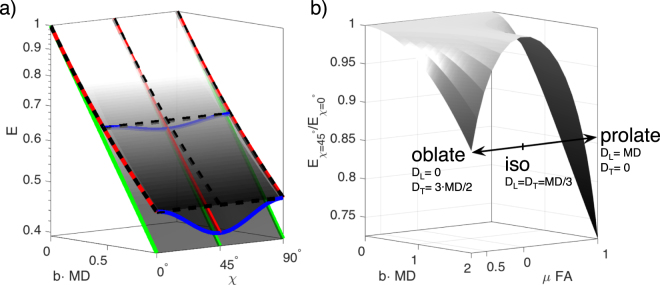
(**a**) An example of signal surfaces for three different distributions of diffusion tensors calculated from Eqn. 10 over a range of *b*-values and ellipticity angles *χ*. MD is the mean diffusivity of the underlying diffusion tensor. The lowest plane highlighted with green lines is a single isotropic domain resulting in a mono-exponential attenuation and no *χ*-modulation. The curved middle plane highlighted with solid red lines over different *b* and solid blue lines over different *χ* was generated from an ensemble of uniformly oriented anisotropic tensors. The top plane highlighted with dashed black lines comes from a distribution of isotropic diffusivities and shows no *χ*-modulation as expected for isotropic compartments. The three planes share the same mean diffusivity indicated by their common initial slope. (**b**) The ratio *E*_*χ*=45_/*E*_*χ*=0_ for *D*_*T*_ – *D*_*L*_ varied to realize different tensor geometries from oblate to prolate with varying *μ*FA. The ratio, corresponding to the depth of the modulation, clearly depends on *μ*FA and tensor geometry.

Figure 2a shows an example of signal attenuations described by Eqs. 10, 11 and 12. Underlying microscopic anisotropy gives a modulation over *χ* with the lowest signal at 45° similar to a DDE experiment with an orthogonal gradient pair. The depth of this *χ*-modulation via the ratio ${E}_{\chi {\mathrm{=45}}^{\circ }}/{E}_{\chi {\mathrm{=0}}^{\circ }}$ depends under our assumptions solely on the difference *D*_*T*_ − *D*_*L*_. Figure 2b shows this ratio for different tensor geometries with different *μ*FA. Oblate geometry corresponds to *D*_*T*_ > *D*_*L*_, prolate to *D*_*T*_ < *D*_*L*_.

## Results

### EP-OGSE on *ex-vivo* brain tissue

**Figure 3:**
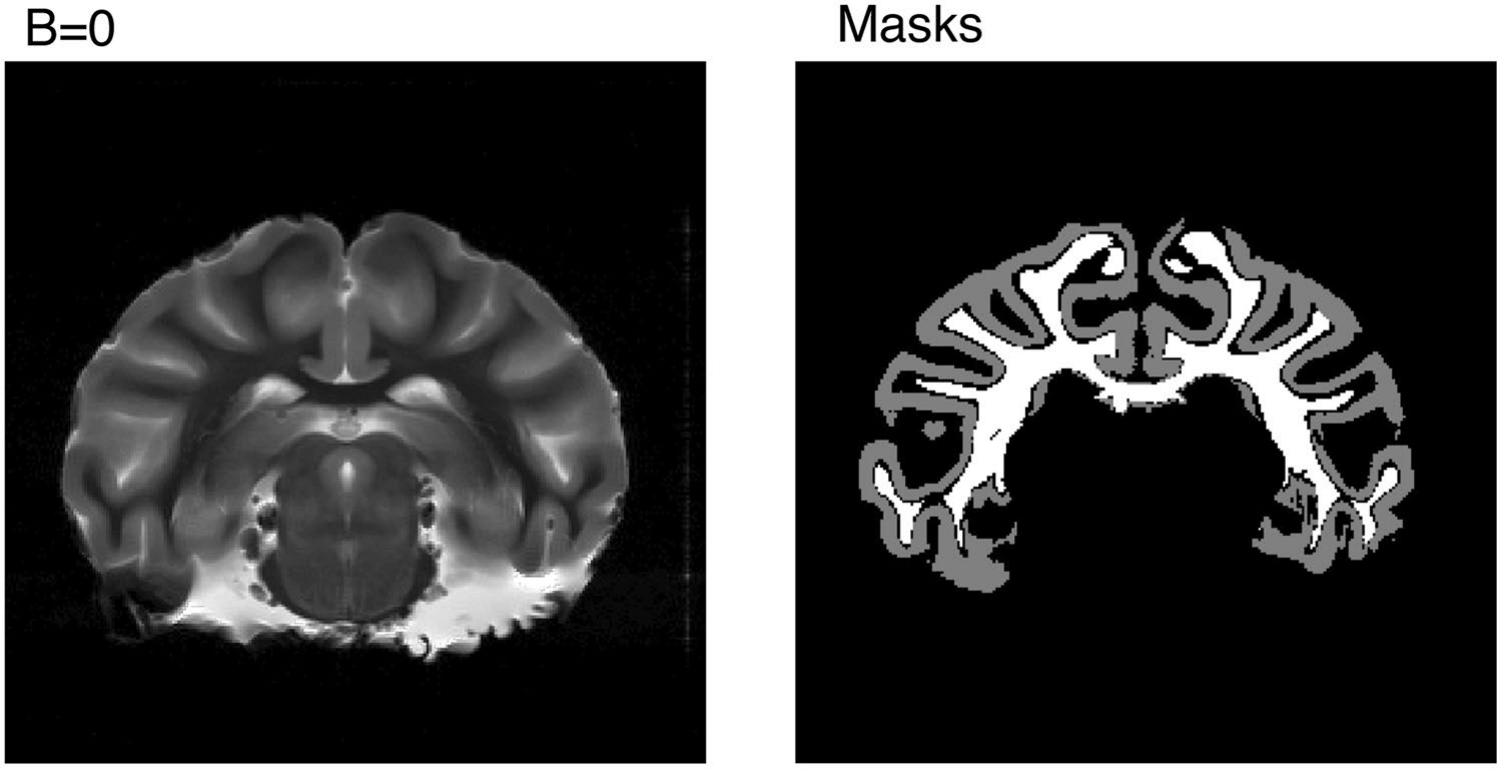
A coronal b = 0 image of the monkey brain and gray and white matter ROIs created from thresholding the b = 0 image. Subcortical structures were excluded by manual delineation.

**Figure 4:**
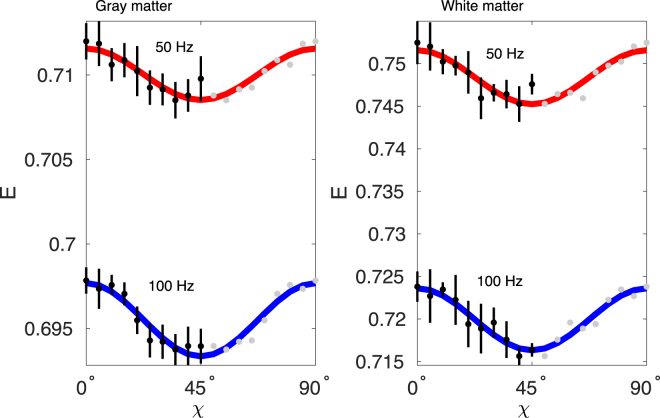
Signal (dots) and fitted model (solid lines) over ellipticity angle *χ* in the gray and white matter ROIs at 50 Hz and 100 Hz. Data were averaged over waveforms with the same eccentricity to achieve a more robust estimate of a uniform distribution and points over 45° are mirrored and redundant and thus plotted in gray. The standard deviation over 6 averages are shown as error bar for each data point. Note that the y-scales are different and that the *χ*-modulation is larger in white matter.

Figure 3 shows a *b* = 0 coronal slice from a vervet monkey used in the experiment and derived masks for gray and white matter. Repetition averaged data normalised to *b* = 0 from the respective regions over different polarization angles and fixed *b* = 0.8 ms/*μ*m^2^ are shown in Fig. 4 and reflect the theoretical predictions for anisotropic substrates (blue curves in Fig. 2a). The baseline signal decreases with frequency as expected from the sigmoidal shape of the diffusion spectrum for restricted diffusion illustrated in Fig. 1d. The fitted model (solid lines in Fig. 4) corresponds well to the measured data with fitted model parameters stated in Table 1, suggesting an increase in both *D*_*L*_ and *D*_*T*_ with frequency with a net decrease in *μ*FA in both gray and white matter. The time dependence in this frequency span is most pronounced in white matter compared to gray matter. Note that a voxelwise map was not possible as the measurements where performed with fixed gradient directions and analyzed over large ROIs to provide rotationally invariance and high SNR.

**Table 1:** Fitted longitudinal and transversal diffusivities *D*_*L*_ and *D*_*T*_ (*μ*m^2^/ms) and microscopic fractional diffusivity *μ*FA from the gray and white matter ROI data shown in Fig. 4. The indicated errors are standard deviations on the parameters calculated from the least squares fit.

Gray matter			**White matter**		
	50 Hz	100 Hz		50 Hz	100 Hz
*D*_*L*_	0.73 ± 0.01	0.83 ± 0.01	*D*_*L*_	0.81 ± 0.02	0.89 ± 0.02
*D*_*T*_	0.28 ± 0.007	0.28 ± 0.005	*D*_*T*_	0.16 ± 0.01	0.19 ± 0.007
*μ*FA	0.54 ± 0.02	0.60 ± 0.01	*μ*FA	0.78 ± 0.03	0.76 ± 0.02

### EP-OGSE Monte Carlo simulations with ellipsoidal restrictions

**Figure 5:**
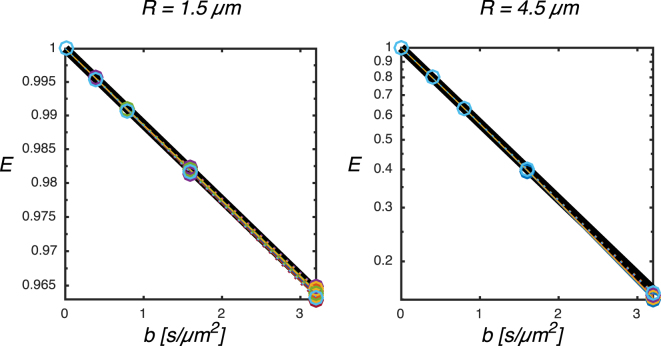
Attenuation curves for spherical restrictions with radii 1.5 and 4.5 *μ*m at 50 Hz for the EP-OGSE measurements in 24 uniformly distributed orientations performed with *χ* = 0° (circles, solid colored lines) and *χ* = 45° (circles, dotted colored lines). The black line indicates the initial monoexponential decay and deviations from this is observed at high *b*-values/gradient strengths as the diffraction limit is approached.

Monte Carlo simulations were performed in ellipsoidal restrictions with 24 uniformly distributed gradient orientations. Biases could be introduced by non-monoexponential signal decay in the individual compartments. To investigate this we plot the log transformed simulated signals in two spherical domains and compare these to the linear attenuation calculated from the lowest *b*-value (Fig. 5). We observe good linearity up to a *b*-value of 1.6 s/*μ*m^2^ with less than 0.2% deviation but up to 1% at 3.2 s/*μ*m^2^. These effects are larger at *χ* = 0° compared to *χ* = 45°. The signal is expected to be rotationally invariant in the spherical restrictions but signal deviations up to 0.4 % at 3.2 s/*μ*m^2^ were observed across the 24 different gradient orientations reflecting the numerical noise levels in our simulation. Only *b*-values up to 1.6 s/*μ*m^2^ where included in the following analysis.

**Figure 6:**
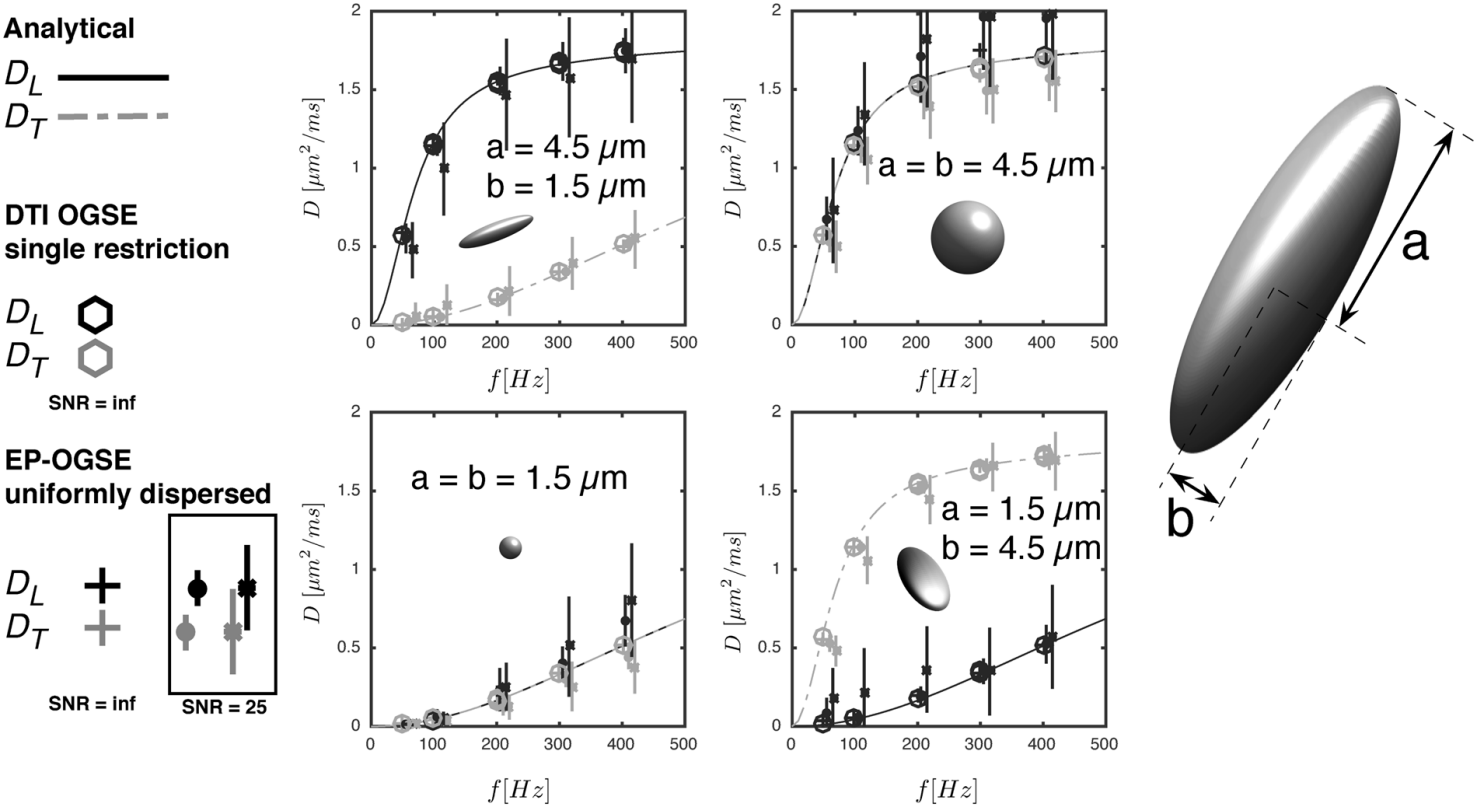
Longitudinal (black) and transversal (gray) temporal diffusion spectra for ellipsoidal restrictions with longitudinal and transversal axes (**a** and **b**) estimated from EP-OGSE Monte Carlo simulations of fully dispersed samples (crosses) compared to estimates from conventional DTI OGSE of single compartments (hexagons). The EP-OGSE estimates were also estimated from noisy data with SNR = 25 using both a maximum b-value of 1.6 s/*μ*m^2^ and 0.6 s/*μ*m^2^ shown as filled dots and asterices. The latter *b*-value is in the range possible up to 100 Hz at a maximum gradient strength of 300 mT/m. The error bars indicate the standard deviation of each estimate. The solid lines are the analytical solutions for spheres with radii a (solid black) and b (gray dashed).

The temporal diffusion spectra from four restrictions with different sizes and shapes are shown in Fig. 6. We here see good agreement between the *D*_*L*_ and *D*_*T*_ calculated from conventional OGSE in aligned compartments and those found by fitting Eqn. 10 to a uniformly distributed powder average (squares vs. crosses in Fig. 6). To investigate the influence of noise, the 96 Monte Carlo datapoints (4 *b*-values and 24 directions) were diluted with random noise from a Gaussian distribution reflecting an SNR of 25 (solid dots with error bars). An additional noise analysis was performed in a simulation with a lower maximum *b*-value of 0.6 s/*μ*m^2^ (asterices with error bars). The results demonstrate that the precision of the estimates improves with *b*-value and the maximum gradient amplitude available. We also see that the diffusion spectra along the individual ellipsoid axes can be modelled well by interpreting *D*_*T*_ and *D*_*L*_ as the frequency dependent diffusivities in spherical restrictions with the same radius^26,49^, but with some deviations for the most eccentric restrictions (lines in the same figure).

## Discussion

We present an experimentally feasible method for detecting the microscopic temporal diffusion spectrum in disordered microscopically anisotropic tissue. DDE benefits from a direct contrast to the microscopic anisotropy as for instance parametrized by *μ*FA^16^. EP-OGSE combines the individual specificities of OGSE with those of DDE and provides specificity to the microscopic temporal diffusion tensor in disperse samples and its derived metrics such as *μ*FA.

### Experiment Frequency dependent diffusion in tissue

Applying EP-OGSE to a fixated monkey brain, we found increases in the modelled *D*_*T*_ in white matter but not in gray matter. Interestingly, we also found increases in *D*_*L*_ both in gray and white matter not predicted by a cylindrical model with free diffusivity along its length axis commonly assumed in biophysical models. This effect could be due to undulations on the length scale of the free diffusion path length of the encoding gradient or structures in the cytoplasm along the axon or in the extracellullar matrix acting as barriers^21,23,50,51^. The data at hand with only two frequency points does not reveal the curvature of the diffusion spectrum but the prospect of isolating the time dependent anisotropic components of a complex tissue environment could potentially isolate interesting features in future experiments. The effective medium theory (EMT) describes the low frequency (long diffusion time) behavior when diffusing spins approach the tortuosity limit^6,22^. The *ω*-dependence predicted by EMT relates to the dimensionality and correlation lengths of a complex geometry. In a disordered 2D lattice, such as the extracellullar space perpendicular to irregularly packed white matter axons, a linear dependence in frequency or 1/*t* is predicted and observed with both OGSE and PGSE^23,24^. In contrast, disorder along 1D structures that may be caused by different varicosities along axons or dendrites relates to a $\sqrt{\omega }$-dependence^21,23^. Even though we were not able to investigate broader frequency ranges our results do support time dependence along and perpendicular to white matter axons, but only along neurites in gray matter. Individual restrictions show a weaker *ω*^2^-dependence which may be a less dominant effect in neuronal tissue with small restriction sizes and low frequencies. This effect could play a larger role in characterization of other tissues as we recently demonstrated in OGSE measurements of myocardial tissue in the heart^52,53^.

### Imperfect orientational distribution

The current experiment was designed to demonstrate the *χ* modulation but has limitations that could be improved in future implementations. Our data do not represent a perfect spherical average of orientations as only one plane was measured (see methods). We believe that this problem was partially mitigated by averaging over a large ROI with a wide distribution of directions but this prevents calculations of voxelwise maps. In a future implementation for voxelwise quantification, the modulation could be estimated from the linear and circular polarisations (*χ* = 0° and *χ* = 45°) acquired in sufficient number of orientations to achieve a robust spherical average similar to previous work using parallel and perpendicular DDE^19^. Interaction with unwanted diffusion weighting from imaging gradients, in particular crusher and slice gradients, might be considered in high resolution settings. Their contributions in the current setting is negligible and would provide only a frequency independent term *B*_*zz*_ in diffusion encoding matrix, perpendicular to the plane of the oscillating gradients^54^.

### Extensions to the single tensor picture

Our analysis includes a single type of disordered local frequency dependent diffusion tensors which may be overly simplistic for many tissue types. Different flavors of the standard model models often relates the multi-exponential signal from individual components with Gaussian or at least mono-exponential behavior. A true biological substrate may be expected to contain a distribution of compartments of varying shape, size and orientations. Given the independent sensitivities of EP-OGSE to variations related to anisotropy and distributions of isotropic diffusivities, we may be able to improve our modelling by adapting approaches in the DDE literature based on for instance cumulant expansion representations of the signal, model based assumptions of the underlying distributions or multidimensional Laplace transforms^19,55,56^. This may also yield other correlation experiments exploring joint distributions of diffusion, diffusion time, chemical shift, exchange, relaxation and magnetization transfer effects^57–61^. Time dependent diffusion implies a non-Gaussian diffusion propagator with non-monoexponential attenuation with respect to the *b*-value^22^. This would violate the simplistic view of the signal as a sum of mono-exponential signal components in Eqn. 12. However, the OGSE signal attenuation is built up by repeated phase encodings with low dephasing amplitude which to a larger degree satisfies the GPA with a mono-exponential decay compared to a PGSE with the same *b*-value^62^. The longer gradient duration of the OGSE experiment also allows for exchange effects to attenuate which could describe the lower kurtosis observed in OGSE data compared to PGSE acquired with the same *b*-value and settings reflecting similar diffusion times^34^. It is yet an open question to what extent the residual non-monoexponential decay in OGSE can be viewed as a mixture of non-exchanging compartments with individual mono-exponential decays. Such effects could arise from either a spread in isotropic diffusivities or an orientational distribution of anisotropic diffusivities. An interesting extension to the current EP-OGSE experiment would be to explore exchange effects at constant *ω*_*m*_ with a varying number of oscillations.

## Simulations

### Agreement with single-compartment OGSE

Our Monte Carlo simulations confirm, under the assumption of axial symmetry, that the EP-OGSE retrieves the same frequency dependent diffusion tensor metrics in samples with isotropic orientational distributions as a DTI analysis of OGSE data on a single substrate. This demonstrates disentanglement of the orientational distribution, a global feature, from the microscopic single compartment diffusivity. We found a good correspondence between the analytical solution for the diffusion spectrum in a sphere and *D*_*L*_ and *D*_*T*_ derived from both EP-OGSE and DTI OGSE in ellipsoidal geometries with the same axial radii. This could be useful for analytical calculation of the signal from arbitrary waveforms without the need of computationally expensive simulations. Estimates from noisy data showed that some bias in *D*_*L*_ and *D*_*T*_ can be expected from data with an experimentally feasible SNR of 25 especially for larger restrictions. Noise sensitivity decreased for the dataset with the highest maximum *b*-value.

### Disentangling global effects

Under the assumption of a single local compartment, linear encoding and Eqn. 11 are sufficient to estimate the underlying anisotropic diffusion spectrum as proposed in previous studies^8,44^. The benefit of multidimensional encoding lies in disambiguating the non-monoexponential attenuation arising from distributions of isotropic diffusivities from that arising from disperse anisotropic diffusion tensors, as illustrated on Fig. 2a: While linear encoding provides the same surface for both kinds of tissue, EP-OGSE provides a *χ*-modulation with anisotropic compartments. Additionally, the depth of this modulation depends directly on *μ*FA as illustrated in Fig. 2b.

### Gradient interactions

The Monte Carlo simulations demonstrated mono-exponentiality up to moderate *b*-values shown in Fig. 5. For the largest *b*-value and large restrictions we indeed observe deviations that can bias the measurement which relies on signal deviations on the order of a few percents. This indicates that the wavelength of the phase encoding wave vector 1/**F**(*t*) approaches the restriction size as can explored in the diffraction patterns at high *q*-values in conventional PGSE^36^. Further, this effect was as expected largest for *χ* = 0° where the gradient amplitude is concentrated to one axis and would thus underestimate the *χ*-modulation in this geometry. Due to the good agreement between the Monte Carlo data and the analytical description of the diffusion spectra we surmise that the dominating non-monoexponential decay can be attributed to anisotropy under the conditions of the simulations. Further spin displacement correlations between the axes may occur in the presence of bulk flow or if the single compartment signals are not well described by the Gaussian phase approximation, for instance due to more complex shapes like curvatures on the length scale of the free diffusion path length over the time frame of the encoding gradients^63,64^.

### EP-OGSE in perspective

#### Similarity with angular DDE

The EP-OGSE sequence has some similarities with the classic angular DDE experiment. However, rather than changing the angle of a second encoding gradient, the relative strengths of two dephased orthogonal gradients are modulated, thus varying the weights of the diffusion encoding axes while fixing their orientations. The off diagonal terms of **B** vanish, excluding signal modulations from non-uniform orientational distributions, while symmetries of the signal over the modulation angle *χ* are introduced.

A similar approach was also recently presented in an angular DDE experiment by Paulsen *et al*. where the relative strengths of two subsequent pulsed field gradient blocks were modulated^43^. This leads to a similar modulation over 90° in anisotropic samples. However, the diffusion is here probed at different (but interdependent) time scales along each axis. A benefit of EP-OGSE is that the probed time scale is constant over *χ* and can be independently modulated by *ω*_*m*_.

Under the assumption of time independent diffusion, fixed encoding axes can also be achieved if multiple encoding gradients deviate from the same symmetry axis with the same but opposed angles or by numerically optimized gradient waveforms^48,65^.

#### Trapezoidal instead of harmonic waveforms

Ianus *et al*. suggested in a simulation study to use an angular DDE experiment with trapezoidal OGSE waveforms for model based estimation of axonal diameters^66^. Our gradient waveform layout with simultaneous gradients has the benefit of maximizing the available time for diffusion weighting on both axes which both increases the maximum diffusion weighting with a factor of two as well as balances the gradient usage over the sequence and corrects for possible gradient imperfections. The encoding gradient waveforms and thus the encoding power spectra, i.e. the filters to the temporal diffusion spectrum, are also maintained along each axis with this configuration at all mixing times deliberately chosen or dictated by the length of the inversion pulses. However, at the expense of lower *b*-value and longer sequence length, separated gradient pairs with constant amplitude decouples the individual encodings and eases the requirement of mono-exponential decay with analytical descriptions of the *b*^2^-term^67,68^.

Trapezoidal oscillating gradients in single directions have been proposed as more efficient than harmonic oscillations used in this study as they provide higher relative spectral encoding amplitude than cosine gradients of equal duration^38,66^, but could in principle also be applied simultaneously. This would however with maximum gradient amplitude be limited to gradient trajectories on a cube and not with uniformly distributed orientations. Experimentally, trapezoidal gradients can also be difficult to achieve in practice at higher frequencies due to hardware limitations^68^. OGSE is in general challenging to perform on clinical systems with limited gradient amplitude and the EP-OGSE method will initially be more suitable for preclinical MRI or diffusion NMR experiments of phantoms or post mortem tissue. With gradient strengths of 80 mT/m or 300 mT/m, reflecting state-of-the-art clinical and experimental human MRI gradient systems the available *b*-value at 100 Hz would according to the trace of Eqn. 4 be on the order of 0.045 and 0.63 s/*μ*m^2^ for two 40 ms gradients in a spin echo sequence. The latter approaches the *b*-value of 0.8 s/*μ*m^2^*μ*m^2^ used in the experiments of this study and was thus included in the simulations. For applications in non-neuronal tissue, for instance in muscle or tumor tissue, larger restriction sizes could be investigated with lower frequency demands and potentially higher *b*-values. Optimal frequency range and SNR requirements should be considered with respect to the expected intrinsic diffusivity and geometry of the restriction of interest.

## Conclusion

EP-OGSE combines OGSE with DDE, offering sensitivity to microscopic anisotropy and the frequency dependent *μ*FA in disperse media of uniformly oriented, axially symmetric diffusion compartments. Monte Carlo simulations show that EP-OGSE retrieves the same single compartment frequency dependent diffusion tensor in disperse media as OGSE in a single aligned compartment. Measurements on *ex-vivo* monkey gray and white matter imply an increase with frequency of transverse as well as longitudinal diffusivity and a net decrease in *μ*FA. The proposed method demonstrates the ability to characterize the temporal diffusion tensor spectrum and provides input for understanding the signal from more general gradient waveforms.

## Methods

### Experiment

EP-OGSE data were acquired using an Agilent 4.7 T preclinical scanner using a quadrature transmit/recieve coil. EP-OGSE gradient waveforms were designed using the CP-OGSE gradient designs described earlier but scaled as described in the theory section^41^. The gradient amplitudes were adjusted numerically to realize a b-value of 0.8 ms/*μ*m^2^ at oscillation frequencies of 50 Hz and 100 Hz with a waveform length of 25 ms repeated on each side of a refocusing pulse in a spin echo sequence. The ellipticity angle *χ* was incremented in 19 steps from 0° to 90° and applied in one plane parallel with the image plane. Maximum gradient strength applied was 500 mT/m. 4 coronal slices were acquired with a 2D spin-echo sequence with a voxel size of 0.23 × 0.23 × 2.5 mm^3^, matrix size of 256 × 256, TE = 71.7 ms and TR = 2.5 s. The protocol was repeated 6 times and every measurement was interleaved with a b = 0 image resulting in 760 image volumes and a total imaging time of 82 hours. To ensure stable sample and RF-coil temperature during different gradient loads, the sample was heated using a conditioned air flow through the bore controlled by a small animal monitoring system (SA Instruments). The temperature was measured on the coil wall 5 cm from the sample and kept at (23 ± 0.1)°C throughout data acquisition. An excised perfusion fixated brain from a 3.5 year old vervet monkey was used for the experiments. Residual formalin from the fixation process was rinsed in phosphate buffered saline (PBS) to enhance T2-relaxation and the brain was submerged in a sealed plastic bag with new PBS using a setup optimised for *ex vivo* DWI described earlier^69^. The post mortem monkey brain specimen was obtained from the Behavioral Science Foundation, St. Kitts and the live animal was socially housed in enriched environments. All procedures for handling and sacrificing the live animal prior to our post mortem experiment were reviewed and approved by the Institutional Review Board of the Behavioral Science Foundation acting under the auspices of the Canadian Council on Animal Care.

### Simulation

Monte Carlo random walk simulations were performed using in house software implemented in Matlab (Mathworks, Natick, MA, USA) optimized with regards to step length and number of walkers^70^. Random walks were performed in axially symmetric ellipsoidal restrictions with all 16 combinations of longitudinal and transversal radii *a* and *b* between 1.5 and 4.5 *μ*m with increments of 1 *μ*m. Time step was 1 *μ*s and the free diffusivity was set to *D*_0_ = 2 *μ*m^2^/ms. The waveform layout was identical to the experiment, but performed with *χ* = 0° and 45° only, 5 frequencies between 50 and 400 Hz and b-values for b = [0 0.4 0.8 1.6 3.2] ms/*μ*m^2^ and b = [0 0.15 0.3 0.45 0.6] with 24 uniformly distributed orientations over the sphere relative to the substrates symmetry axes.

### Analysis

The analyses were performed with in house software implemented in Matlab (Mathworks, Natick, MA, USA). Masks for gray and white matter ROIs were created by thresholding the b = 0 images. All diffusion weighted images were normalized to its subsequent b = 0 image and the data were averaged over the two ROIs. To eliminate effects of global anisotropy, the data were also averaged symmetrically around *χ* = 45°. Subcortical structures were excluded using a manually drawn mask. A slight evaporation of PBS was observed during the experiment resulting in a shift in intensity in partial volume voxels. To only include data with consistent tissue fractions we excluded voxels with more than 5% signal variation in the b = 0 images over the experiment. B-matrices were calibrated for each gradient waveform to achieve a constant signal attenuation on a water ROI corresponding to a free diffusivity of *D*_0_ = 2 *μ*m^2^/ms reflecting the free diffusion coefficient at room temperature.

Simulation data were analysed in two ways. First, the compartment diffusion tensor spectrum for each substrate was estimated from data with linear polarisation using linear regression. Second, data were averaged over all gradient directions for each frequency, substrate and polarisation to emulate an isotropic orientational distribution.

Eqn. 10 was fitted to both the averaged experimental and simulated data with respect to *D*_*L*_ and *D*_*T*_ using non-linear least squares fitting. Simulated data were compared with the analytical descriptions for the diffusion spectrum in spherical restrictions^26^. The error intervals of the experimentally determined *D*_*L*_ and *D*_*T*_ were calculated as the diagonals of $\sqrt{{({J}^{T}J)}^{-1}{\sigma }_{s}}$ where *J* is the estimated Jacobian provided by Matlab in the least squares fit and *σ*_*s*_ is the variance of the residuals. Error propagation was further assessed in the Monte Carlo data with random Gaussian noise added to the EP-OGSE data reflecting an SNR of 25 in the individual datapoints. Mean and standard deviations of the parameter fits were calculated over 100 repetitions.

### Data availabillity

The datasets generated during and/or analyzed during the current study are available from the corresponding author on reasonable request.
